# Using LOTOS for Formalizing Wireless Sensor Network Applications

**Published:** 2007-08-13

**Authors:** Nelson Souto Rosa, Paulo Roberto Freire Cunha

**Affiliations:** Universidade Federal de Pernambuco, Centro de Informática, Recife, BrazilE-mails: nsr@cin.ufpe.br, prfc@cin.ufpe.br

**Keywords:** Wireless Sensor Network Applications, LOTOS, Formalization

## Abstract

The number of wireless sensor network (WSN) applications is rapidly increasing and becoming an integral part of sensor nodes. These applications have been widely developed on TinyOS operating system using the nesC programming language. However, due to the tight integration to physical world, limited node power and resources (CPU and memory) and complexity of combining components into an application, to build such applications is not a trivial task. In this context, we present an approach for treating with this complexity adopting a formal description technique, namely LOTOS, for formalising the WSN applications ‘behaviour. The formalisation has three main benefits: better understanding on how the application actually works, checking of desired properties of the application's behaviour, and simulation facilities. In order to illustrate the proposed approach, we apply it to two nesC traditional applications, namely BLink and Sense.

## 1. Introduction

The number of wireless sensor network (WSN) applications is rapidly increasing and becoming an integral part of sensor nodes. Those applications that were originally military ones, have been spread out through several areas, including environment, habitat, disaster monitoring, healthcare applications, home automation, and traffic control [1, 2].

WSNs applications have been widely developed on TinyOS operating system [3] and written in nesC programming language [4]. In fact, it is worth observing that most the current sensor network hardware systems are TinyOS compatible. Unlike traditional embedded operating systems, TinyOS does not provide real-time properties, as WSN applications usually do not need of this kind of characteristic. Additionally, TinyOS applications are developed as a set of wired components that work to execute a task.

Despite the current support to develop WSN applications provided by TinyOS, the tight integration to physical world, limited node power and resources (CPU and memory), and complexity of combining components into an application become the construction of an application a non trivial task. In particular, the usual great number of components that compose the application together the way they interact put some challenges on understanding how the application actually works.

In this context, we present an approach for treating with the complexity of building WSN applications through adopting a formal description technique, namely LOTOS [5], for formalising their behaviour. The formalisation has three main benefits: better understanding of applications behaviour, checking of desired properties of the application, and possibility of simulating applications prior to build them.

The choice of LOTOS has been based on some facts. Firstly, it is a ISO standard specially designed for describing the behaviour of concurrent systems like nesC applications. Secondly, the way LOTOS applications are structured (hierarchically) is very similar to the way the applications are organised in nesC. Finally, the expressiviness of LOTOS enables us to specify the application in many different levels of abstraction, which facilitates to treat with the complexity of nesC applications.

Current approaches for building WSN applications includes those to formally design embedded systems [6], a practical guide for building sensor applications [7] and an approach for prototyping applications in BTNodes [8]. Despite the fact that WSN applications are also embedded, the absence of real-time requirements changes significantly the way these applications are formalised. For example, time issues are not explicitly considered in WSN applications. Meanwhile, the SNACK kit concentrates on contruction of applications without adopting a formal approach. Finally, the construction of applications in BTNodes simply addresses the facilities provided by the hardware itself for building them.

In addition to the this introduction, this paper is organised into four sections. Section 2 introduces basic concepts of LOTOS and TinyOs prior to present the proposed formalisation approach. Next, Section 3 presents the proposed approach to model TinyOS applications in LOTOS. Section 4 shows how the modelling approach may be applied to two well-known TinyOS applications, namely Blink and Sense. Finally, last section presents conclusions and some future work.

## 2. Basic Concepts

Prior to present our approach to formalise WSN applications, next subsections introduce basic concepts of LOTOS and TinyOS.

### 2.1. LOTOS

A LOTOS (Language Of Temporal Ordering Specification) [5, 9] specification describes a system through a hierarchy of active components or processes. A process is an entity able to realise non-observable internal actions and to interact with other processes through externally observable actions. The unit of atomic interaction among processes is called an event. Events correspond to a synchronous communication that may occur among processes able to interact with one another. Events are atomic, in the sense that they happen instantaneously and are not time consuming. The point where an event interaction occurs is known as a port. Such event may or may not actually involve the exchange of values. A non-observable action is referred to as an internal action or internal event. A process has a finite set of ports that can be shared.

An essential component of a specification or process definition is its behaviour expression. A behaviour expression is built by applying an operator (e.g., parallel operator “| |”; to other behaviour expressions. A behaviour expression may also include instantiations of other processes, whose definitions are provided in the “ where” clause following the expression [5]. Next, we present the LOTOS specification of a simple client-server system:

(1)
**specification** ClientServer [request,reply]:**noexit**
(2) 
**behaviour**
(3) 
Client [request,reply] | |Server [request,reply]
(4) 
**where**
(5)  
**process** Client [request,reply]: **noexit** :=
(6)   
request; reply; Client [request, reply]
(7) 
**endproc**
(8) 
**process** Server [request,reply]:**noexit**:=
(9)  
**hide** processRequest **in**
(10)   
request;
(11)   
processRequest;
(12)   
reply;
(13)   
Server [request, reply]
(14)  
**endproc**
(15) 
**endspec**

The top-level specification (3) is a parallel composition (operator '‖') of the processes Client and Server i.e., every action externally observable executed by the process Client must be synchronised to the process Server. The process Client (5) performs two actions, namely request and reply (6), and then reinstantiates. The action-prefix operator (‘;’) defines the temporal ordering of the actions request and reply (the action request occurs before the action reply) in the Client. Informally, the Server (8) receives a request (10), processes it (11) and then sends a reply (12) to the process Client.

It is worth pointing out that LOTOS specifications may be compared in order to check their behavioural equivalences such as strong equivalence, observational equivalence and safety equivalence. All of them are checked through the CADP Toolbox*.

### 2.2. TinyOS

TinyOS [3] is the first operating system specially designed for wireless sensor networks. Basic in TinyOS is the fact that those applications are event-driven in such way that when an event occurs a handler may post a task that is scheduled by the TinyOS Kernel some time later. TinyOS applications are written in nesC.

nesC (network embedded system C) [4] is a structured component-based dialect of C specially designed for building embedded systems like WSN applications. An application is nesC consists of a set of components linked together with bidirectional interfaces that serve as access points to the component. Two kinds of components may be defined in nesC: *configuration* and *module*. A configuration simply defines how components are put together (wired), whilst modules represent the implementation itself. A component *provides* and *uses* interfaces that declare a set of functions (*commands*) the component must implement and functions (*events*) that the user of the interface must implement. Every nesC application is described by a top-level configuration that wires components. Additionally, two basic elements of TinyOS applications include: the *Main* component that must be implemented in every application; and the standard interface named *StdControl* used to initialise/start/stop TinyOS components, which includes three operations: *start*, *init* and *stop*.

## 3. Modelling WSN Applications in LOTOS

The proposed approach for specifying WSN applications in LOTOS considers some basic principles that act as a guideline in the specification process. Firstly, as a specification language, LOTOS is used to describe the behaviour of nesC applications. Hence, typical constructions of imperative language such as loop, decision and assignment commands are not present in the specification. Secondly, the specification concentrates mainly on identifying and modelling interactions between components. In particular, interactions in LOTOS are always synchronous. Thirdly, the LOTOS state-oriented specification style [10] is extensively adopted. In this style, the whole system (or part of the system) is considered a single resource whose internal space is explicitly defined as a set of states and alternative sequence of interactions.

Following these principles, the LOTOS modelling process is carried out in four main steps:

1. To identify the nesC components (modules and configurations) that made up the application. These components become LOTOS processes at the end of this step;
2. To identify how components (modules and configurations) found in Step 1 are wired and which interfaces are used to connect them. The way the components are wired defines how the LOTOS processes are composed, whilst the interfaces become synchronisation ports in LOTOS;
3. To identify operations (commands and events) of each interface identified in Step 2. For each interface, it is defined a LOTOS ADT (Abstract Data Type) whose operations represent the nesC commands and events; and
4. To identify interactions inside nesC modules' implementation. After identifying the modules in Step 1, their composition in Step 2, and the operations available to be invoked by each module in Step 3, it is time to precisely identify the order the operations are invoked inside the modules. When a nesC operation is called, this indicates that an interaction takes place between the nesC modules. Hence, in LOTOS, these interactions represent synchronisation events between two processes.

Next sections present how nesC first-order elements identified in each previous step are modelled in LOTOS.

### 3.1. Interface

As mentioned before, nesC applications are built out of components with well-defined interfaces, which represent points of access to the components. The interaction between components occurs through the invocation of functions in the interface. In LOTOS, processes interact through their synchronisation ports (see Section 2.1). In this way, the nesC interface is defined as a LOTOS synchronisation port e.g., if a component provides three interfaces it has three ports. Furthermore, as LOTOS ports have no direction (there is not the notion of output/input ports), provided and used interfaces are simply modelled as ports. The actions that occur in the port give an idea if the interface is a used or provided interface.

**Figure 1.**
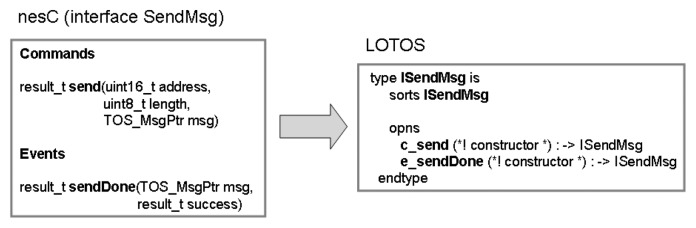
nesC interface in LOTOS

As mentioned in Section 2.2, each nesC interface has a set of functions (commands and events). As we are only interested in the module interactions or temporal ordering of events (not in their functionality), these functions are defined as LOTOS operations of an abstract data type (ADT) defined for each interface. In this way, the nesC interface <*interface-name*> leads to the definition of the LOTOS ADT “I”<*interface-name*>. The command <*command-name*> and event <*event-name*> are defined as type operations “c_”<*command-name*> and “e_”<*event-name*>, respectively. In order to illustrate this approach, the interface SendMsg is presented in Figure 1.

In this example, the nesC interface SendMsg is modelled as the LOTOS abstract data type ISendMsg, whilst its command send and event sendDone are defined as the operations c_send and c_event of type ISendMsg. As mentioned before, the semantics of the operation together their input/output parameters have no meaning in LOTOS specification.

### 3.2. Module

**Figure 2.**
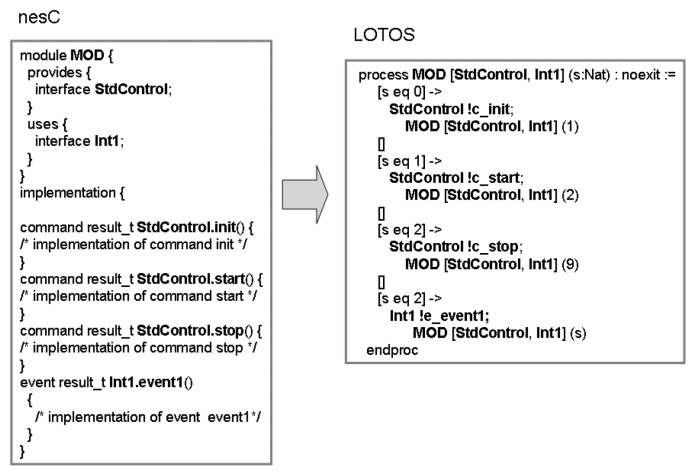
nesC module in LOTOS

A module is a first-class element of nesC language and has of two basic parts: the set of interfaces provided and used by the module; and the implementation. The interfaces are modelled as defined in Section 3.1. Meanwhile, as the basic abstraction in LOTOS is a process, the nesC module is specified as a LOTOS process (see Figure 2). The implementation of each function (command or event) is defined in the behaviour part of the process using the LOTOS choice operator ([ ]): each command/event defines an option in the choice.

Modules that implement the StdControl interface (see Section 3.1) are defined using the state-oriented specification style. In this case, four states are defined: the module initiates in an initial state (state 0) prior to execute the operation StdControl.init; the second state (state 1) is reached when the module is initialised but not started yet (after the execution of StdControl.start); the state when the module is already initialized and ready to execute the functions defined in the interface (state 3); and finally, the last state (state 9), when the module has been stopped after the execution of the function StdControl.stop. Being reached the state 9, the module is not able to execute any other action.

It is possible to observe in the nesC programming that this module interacts with other modules (defined in the configuration where it is placed) through the interfaces StdControl (provides) and Int1 (uses). These interfaces becomes LOTOS synchronisation ports. The interface StdControl has three operations, namely init, start, and stop (renamed to c_init, c_start and c_stop, respectively), defined as choices in the LOTOS choice operator ( [ ]). The interface Int1 is used by this module, which means that the module must implement the events defined in the interface. In this particular case, only the event event1 is defined. Finally, as this module implements the StdControl interface, the state-oriented LOTOS style has been used.

The LOTOS specification ahead expresses that if process MOD is in the state 0 ( [s eq 0]) then a synchronisation action may occur in the port StdControl if the value c_init is offered ( StdControl !c_init). Then the module MOD is instantiate and the new state is set to 1.

(1) $$\begin{array}{cc} \left[s\;eq\;0\right]-> & \\ & \begin{array}{l} \text{StdControl}\;!\;c\_\text{init};\\ \text{MOD}\;\left[\text{StdControl},\;\text{Intl}\right] \end{array} \end{array}$$

### 3.3. Configuration

A configuration is a first-class element of nesC that defines used and provided interfaces, the components that made up it and the way they are wired (implementation). A configuration is present in a nesC application in two situations: the initial configuration of an application; or part of another configuration. In the first case, the configuration is modelled as the LOTOS top-level process in the process hierarchy, which is defined as “specification” (see Section 2.1). In the second case, it is simply modelled as a LOTOS process. In both cases, the module's implementation defines how the modules that made up the configuration are wired and the interfaces used to connect them.

If two modules within the configuration are wired through an interface, it means that they interact each other. Hence, these two modules are placed in a LOTOS parallel composition with synchronisation (according to Step 2, each interface becomes a synchronisation port). Otherwise, the parallel interleaving LOTOS operator (| | |) is used to model the situation when two components are not wired.

In order to illustrate these elements, the specification ahead presents a nesC configuration:

configuration **CONF** {
}
implementation { 
components **Mod1**, **Mod2**, **Mod3**, **Mod4**; 
Mod1.Int1 −> Mod3.Int1; 
Mod1.Int1 −> Mod2.Int1; 
Mod2.Int2 −> Mod3.Int2; 
Mod2.Int3 −> Mod4.Int3;
}

Its respective LOTOS specification is as follows:

(1)
specification **CONF** [Int1, Int2, lnt3] : noexit
(2)
behaviour
(3) 
Mod1 [lnt1]
(4)  
|[lnt1]|
(5) 
Mod2 [lnt1, Int2, Int3]
(6)   
|[Int1, Int2, lnt3]|
(7) 
(Mod4 [Int1] | | | Mod3 [Int1, lnt3];
(8)
where
(9) 
process **Mod1** [lnt1]: noexit
(10) 
:= (* behaviour specification *) endproc
(11) 
process **Mod2** [lnt1, Int2, Int3]: noexit
(12) 
:= (* behaviour specification *; endproc
(13) 
process **Mod3** [lnt1,Int3]: noexit
(14) 
:= (* behaviour specification *; endproc
(15) 
process **Mod4** [lnt1]: noexit
(16) 
:= (* behaviour specification *; endproc
(17)
endspec

The nesC configuration CONF is defined as the LOTOS top-level specification (line 1) and consists of four components: Mod1 (line 9), Mod2 (line 11), Mod3 (line 13) and Mod4 (line 15). According to the proposed approach, each nesC component is defined as a LOTOS process. These components are wired according to the nesC implementation clause: Mod1 is wired to Mod3/Mod2 through the interface Int1; Mod2 is wired to Mod3 through Int2; and Mod2 is wired to Mod4 through Int3. In LOTOS, it means that Mod1 synchronises simultaneously with Mod2 and Mod3 in port Int1 (line 4); Mod2 synchronises with Mod1 in the port Int1 (line 4), with Mod3 in the port Int2 (line 6) and with Mod4 in the port Int3 (line 6); and Mod4 and Mod3 are not connected ( Mod4 [Int3] ||| Mod3 [Int2,Int1]).

## 4. Adopting the Modelling Approach

In order to illustrate the proposed modelling approach, two traditional applications of the TinyOS have been modelled, namely Blink and Application. Both of them are detailed in [3].

### 4.1. Blink Application

**Figure 3.**
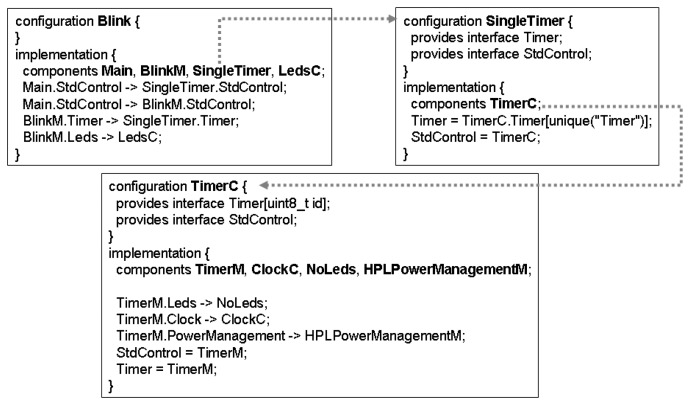
nesC BLink application

Blink is a basic application that toggles the leds on the mote on every clock interrupt. The clock interrupt is scheduled to occur every second. Figure 3 shows the configurations ( BLink, SingleTimer and TimerC) that make up the whole application. The modules can be identified in the clauses “components” within the configurations.

According to Section 3, the first step in the modelling process is to identify the modules and configurations that compose the application. The configuration Blink is modelled as the top-level specification (line 1) and it is defined as a parallel composition (lines 4-10) of four components: module Main (line 12), module LedsC (line 14), module BlinkM (line 16) and configuration SingleTimer (line 18). As can been seen in Figure 3, the component SingleTimer (18) is a configuration that only contains the configuration TimerC (line 19). TimerC includes four components (lines 23-30): module TimerM (32), module NoLeds (35), configuration ClockC (37), module HPLPowerManagementM (line 42). The configuration ClockC only contains the module HPLClock (not shown in specification for lack of space).

As the components have been defined, next step must identify how they are wired and their interfaces (see Figure 3). According to Section 3, nesC interfaces are modelled as LOTOS synchronisation ports. The module Main is wired to SingleTimer ( Main.StdControl −> SingleTimer.StdControl) and BLinkM ( Main.StdControl −> BLinkM.StdControl) through StdControl (line 5); BlinkM is connected to both LedsC and SingleTimer through Leds and Timer, respectively (line 7); TimerM is connected to NoLeds through Leds (line 25); TimerM and ClockC through Clock (line 25); and TimerM to HPLPowerManagementM through PowerManagement (line 25). It worth observing that LedsC and SingleTimer are not connected (line 9;; and NoLeds, ClockC and HPLPowerManagementM are also not wired (lines 27 and 29.



(1)
specification **Blink** [Timer,Leds,StdControl]:noexit
(2)
(* Abstract Data Type definition *)
(3)
behaviour
(4) 
Main [StdControl]
(5)  
| [StdControl] |
(6)
BlinkM [StdControl, Leds, Timer] (0)
(7) 
|[Leds, Timer, StdControl]|
(8)
(LedsC [Leds]
(9)   
| | |
(10)  
SingleTimer [Timer, StdControl];
(11)
where
(12) 
process **Main** [StdControl] : noexit
(13) 
:= (* behaviour of Main *; endproc
(14) 
process **LedsC** [Leds] : noexit
(15) 
:= (* behaviour of LedsC *; endproc
(16) 
process **BlinkM** [StdControl,Leds,Timer](s:Nat) :
(17) 
noexit := (* behaviour of BLinkM *; endproc
(18) 
process **SingleTimer** [Timer, StdControl]:noexit:=
(19)  
TimerC [Timer, StdControl]
(20)  
where
(21)   
process **TimerC** [Timer, StdControl] : noexit :=
(22)    
hide Leds, Clock, PowerManagement in
(23)    
TimerM [Timer, StdControl, Leds,
(24)      
Clock, PowerManagement] (0;
(25)     
|[Leds, Clock, PowerManagement]|
(26)    
(NoLeds [Leds]
(27)      
| | |
(28)    
ClockC [Clock]
(29)      
| | |
(30)    
HPLPowerManagementM [PowerManagement];
(31)    
where
(32)     
process **TimerM** [Timer,StdControl,Leds,
(33)      
Clock,PowerManagement](s:Nat;:noexit
(34)     
:= (* behaviour of TimerM *; endproc
(35)     
process **NoLeds** [Leds] : noexit
(36)     
:= (* behaviour NoLeds *; endproc
(37)     
process **ClockC** [Clock] : noexit
(38)     
:= (* behaviour of ClockC *; endproc
(39)     
process **HPLClock** [Clock,StdControl](s:Nat):
(40)     
noexit
(41)     
:= (* behaviour of HPLClock *; endproc
(42)     
process **HPLPowerManagementM** [PowerManagement]:
(43)     
noexit
(44)     
:=(*behaviour of HPLPowerManagementM*;endproc
(45)
endspec

In the Step 3, command and events of the interfaces must be identified. For each interface, a LOTOS abstract data type is defined and each type operation models a command/event. Next, we present the LOTOS specification of the nesC interface StdControl (see Section 2.2):

type IStdControl is
sorts IStdControl
opns 
c_init (*! constructor *) : −> IStdControl 
c_start (*! constructor *) : −> > IStdControl 
c_stop (*! constructor *) : −> IStdControl
endtype

This interface ( IStdControl) has three commands and no events: c_init initializes the component and its subcomponents; c_start starts the component and its subcomponents; and c_stop stops the component and pertinent subcomponents.

Finally, in the last step, through observing the implementation of the modules, it is possible do precisely define which commands and events are called inside them. For example, the nesC implementation of module BLinkM

module **BlinkM** { 
**provides** { 
  interface **StdControl**;} 
**uses** {  
interface Timer;  
interface Leds;}} 
**implementation** {  
command result_t **StdControl.init**() {   
call Leds.init(;;   
return SUCCESS; }  
command result_t **StdControl.start**() {   
return call Timer.start (TIMER_REPEAT, 1000;;}  
command result_t **StdControl.stop**() {   
return call Timer.stop(;;}  
event result_t **Timer.fired**() {   
call Leds.redToggle(;;   
return SUCCESS;}
}

The corresponding LOTOS specification is defined as follows:

(1)
process **BlinkM** [StdControl, Leds, Timer] (s:Nat)
(2)
: noexit :=
(3) 
[s eq 0] −> > **StdControl !c_init**;
(4)  
Leds !c_initLeds;
(5)   
BlinkM [StdControl, Leds, Timer] (1;
(6) 
[ ]
(7) 
[s eq 1] −> **StdControl !c_start**;
(8)  
Timer !c_startTimer;
(9)  
BlinkM [StdControl, Leds, Timer] (2;
(10) 
[ ]
(11) 
[s eq 2] −> **StdControl !c stop**;
(12)  
Timer !c_stopTimer;
(13)  
BlinkM [StdControl, Leds, Timer] (9;
(14)
[ ]
(15)
[s eq 2] −> **Timer !e fired**;
(16)  
Leds !c_redToggle;
(17)  
BlinkM [StdControl, Leds, Timer] (s;
(18)
endproc

The module BlinkM provides the interface StdControl [8](lines 3, 7, 11) and uses both interfaces Leds (lines 4 and 16) and Timer (lines 8 and 12). According to Section 3.2, as this module implements the StdControl interface it is defined using the state-oriented specification style. The states and their changes can be seen in line 5 (from state 0 to state 1), line 9 (from state 1 to state 2), line 13 (from state 2 to state 9).

After being completely specified, several simulations have been carried out using the CADP ToolBox in order to validate the specification. Additionally, two properties have been also checked against the specification: deadlock and livelock. Next, we present the CADP output of checking deadlock freedom:

<INITIAL STATE>
“STDCONTROL !C_INIT”
“LEDS !C_INITLEDS”
“I” (STDCONTROL [208]) /* EXECUTION OF INIT */
“I” (STDCONTROL [208]) /* EXECUTION OF START */
“I” (STDCONTROL [208]) /* EXECUTION OF STOP */YEAR = {2004}
<DEADLOCK>

This sequence indicates the presence of a deadlock. It was expected as the function StdControl.stop leads the state machine of the module that implements the StdControl interface to a state (state 9) where it is not possible to execute any further action. In fact, this behaviour reflects the nesC implementation, which also has a similar deadlock. Additionally, no livelock sequence was found in the specification.

### 4.2. Sense Application

Sense is an application that periodically samples the photo sensor and displays the highest 3 bits of the raw ADC light reading to the leds, with red being the most signficant bit and yellow being the least significant bit.

**Figure 4.**
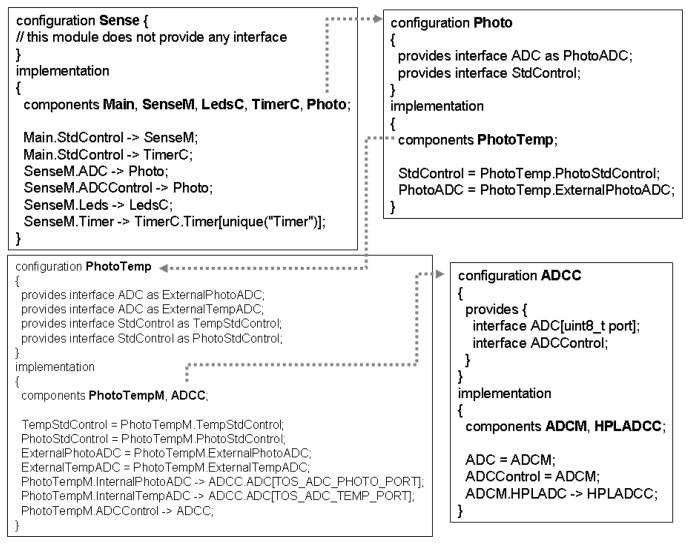
nesC Sense application

Similarly to the BLink, the steps defined in Section 3 have also been followed in the specification of Sense application. Hence, the modules and configurations that compose the Sense application were initially identified (see Figure 4): module Main (line 16), module SenseM (line 18), module LedsC (line 21), configuration TimerC (the same as in the Blink application), and configuration Photo (line 25). The configuration Photo only includes a module, namely PhotoTemp, which is a configuration. PhotoTemp is composed by the module PhotoTempM and the configuration ADCC. Finally, the configuration ADCC is made up of modules ADCCM and HPLADCC.

As the modules and configurations have been identified, it is necessary to defiine how they are wired. For simplicity, the specification ahead only shows the top-level specification Sense, in which we have the following connections: Main to SenseM and TimeC through StdControl (line 6); SenseM to LedsC through Leds (line 8); SenseM to TimerC through Timer (line 8); SenseM to Photo through ADC (line 8), ADCControl and StdControl (line 8). LedsC, TimerC and Photo are not connected (lines 11 and 13).



(1)
specification **Sense** [Timer, Leds, StdControl,
(2)     
ADC, ADCControl] : noexit
(3)
(* Abstract Data Type behaviour *)
(4)
behaviour
(5) 
Main [StdControl]
(6)  |
[StdControl]|
(7) 
SenseM [StdControl,Leds,Timer,ADC,ADCControl](0;
(8)  
|[Leds, Timer, StdControl, ADC, ADCControl]|
(9)
(
(10) 
LedsC [Leds]
(11)  
| | |
(12) 
TimerC [Timer, StdControl]
(13)  
| | |
(14) 
Photo [ADC, ADCControl, StdControl];
(15)
where
(16) 
process **Main** [StdControl] : noexit
(17) 
= (* behaviour of Main *; endproc
(18) 
process **SenseM** [StdControl, Leds, Timer,
(19)      
ADC,ADCControl](s:Nat;:noexit
(20) 
= (* behaviour of SenseM *; endproc
(21) 
process **LedsC** [Leds] : noexit :=
(22) 
:=(* behaviour of LedsC *; endproc
(23) 
process **TimerC** [Timer, StdControl] : noexit
(24) 
:=(* behaviour of TimerC *; endproc
(25) 
process **Photo**[ADC,ADCControl,StdControl]:noexit
(26) 
:=(* behaviour of Photo *; endproc
(27)
endspec

As the main component of the application, the module SenseM is defined as follows:

(1)
process SenseM [StdControl, Leds, Timer,
(2)     
ADC, ADCControl](s:Nat;:noexit:=
(3) 
[s eq 0] −>
(4) 
StdControl !c init;
(5) 
Leds !c initLeds;
(6) 
ADC !c init;
(7) 
SenseM [StdControl,Leds,Timer,ADC,ADCControl](1;
(8)
[ ]
(9)
[s eq 1] −>
(10) 
StdControl !c_start;
(11) 
Timer !c_startTimer;
(12) 
SenseM [StdControl,Leds,ADC,Timer,ADCControl](2;
(13)
[ ]
(14)
[s eq 2] −>
(15) 
StdControl !c_stop;
(16) 
Timer !c_stopTimer;
(17) 
SenseM [StdControl,Leds,ADC,Timer,ADCControl](9;
(18)
[ ]
(19)
[s eq 2] −>
(20) 
Timer !e_fired;
(21) 
ADCControl !c_getData;
(22) 
ADCControl !e_dataReady;
(23) 
SenseM [StdControl,Leds,ADC,Timer,ADCControl](s;
(24)
endproc

As this module implements the StdControl interface, it is also defined using the state-oriented style (states 0, 1, 2, 9). For example, when the process SenseM is in the state 0 (line 3), the only possible action is StdControl ! c_init (line 4). Meanwhile, when the process reaches the state 9 (line 17) after being stopped (line 15), no further actions are possible. An interface that must be mentioned is the ADC:

type IADC is 
sorts IADC 
opns c_getData
(*! constructor *) : −> IADC  
c-getContinuosData
(*! constructor *; : − > IADC  
e_dataReady
(*! constructor *; : − > IADC
endtype

The properties deadlock and livelock have also been checked against this specification. As expected, this specification has a deadlock, which it was also expected, as the nesC application itself stops in a deadlock situation. The sequence that leads to the deadlock is shown in the following:

<INITIAL STATE>
“STDCONTROL !C_INIT”
“LEDS !C_INITLEDS‘
“I”(STDCONTROL [244]) /* init operation */
“I” (STDCONTROL [244]) /* START OPERATION */
“I” (STDCONTROL [244]) /* STOP OPERATION */
<DEADLOCK>

Finally, it is worth observing that the modules and configurations associated to TimerC ( TimerM, NoLeds, ClockC, HPLPowerManagementM, HPLClock) were already present in the BLink application. Due to the approach defined to structure the LOTOS specification, these modules were integrally reused in the Sense specification and no further change was necessary to reuse them.

## 5. Conclusion and Future Work

This paper has proposed an approach useful to formalise the behaviour of WSN applications in LOTOS. The approach consists of moddelling nesC and TinyOS elements in LOTOS. In order to carry ou this task, we defined a set of specification steps together an approach to specify the main concepts of nesC in LOTOS, namely interfaces, modules and configurations.

The approach for formalising nesC applications has some interesting benefits to those who build WSN applications in nesC. Firstly, the specification process improves the understandability of the nesC application itself. Secondly, the emphasis on the interaction of components (instead their functionality) in the LOTOS specification provides a better understanding of the way the components collaborates. Thirdly, the strategy adopted to specify the nesC applications favoured the high reuse of module specifications. Finally, the formalisation allows us to check useful properties of the specification.

We are now extending the proposed set of abstractions including more sophisticated communication and concurrent elements. Meanwhile, it is also planned to include the specification of middleware services in such way that composition constraints may also consider middleware service composition. Another interesting future work that is now being devised refers to the modelling of the operating system itself, which acts as an engine for executing the application.

## Footnotes

* http://www.inrialpes.fr
